# Sjogren’s Syndrome Presenting With Proximal Myopathy Due to Osteomalacia Complicating Renal Tubular Acidosis: A Case Report

**DOI:** 10.7759/cureus.82206

**Published:** 2025-04-13

**Authors:** Mohammad Syedul Islam, Quazi Mamtaz Uddin Ahmed, Farzana Ahmed, Md Ashraf Uddin, Naznin Naher

**Affiliations:** 1 Department of Internal Medicine, Bangabandhu Sheikh Mujib Medical University, Dhaka, BGD; 2 Department of Pediatrics, Marks Medical College and Hospital, Dhaka, BGD

**Keywords:** distal renal tubular acidosis, fracture neck of femur, osteoporosis, proximal myopathy, recurrent hypokalemic quadriparesis

## Abstract

Primary Sjögren’s syndrome (pSS) is typically associated with dryness of the eyes and mouth, but it can also involve other organs, including the lungs, kidneys, nervous system, and joints. Among its less common manifestations is distal renal tubular acidosis (dRTA), which can lead to metabolic acidosis, hypokalemia, and bone-related complications due to chronic acid-base imbalance. We report the case of a 42-year-old woman with a four-year history of recurrent hypokalemic quadriparesis, who recently developed progressive difficulty walking over the past two months, severely limiting her mobility. Laboratory investigations revealed a normal anion gap metabolic acidosis and elevated urine pH, consistent with dRTA. Further evaluation confirmed a diagnosis of pSS with objective evidence of glandular involvement. Imaging and biochemical findings supported the presence of osteomalacia secondary to dRTA. This case highlights a rare and often overlooked complication of pSS. Timely diagnosis and appropriate management are crucial to preventing long-term disability and improving patient outcomes.

## Introduction

Primary Sjögren’s syndrome (pSS) is a chronic autoimmune disorder marked by lymphocytic infiltration of exocrine glands, resulting in persistent inflammation and hallmark symptoms such as dry eyes and dry mouth [1]. Beyond the exocrine glands, mucosal dryness can extend to other surfaces, including the airways, gastrointestinal tract, and vagina, contributing to the broader clinical picture of sicca syndrome. pSS is also known for its potential to affect multiple organ systems, with extraglandular manifestations classified as either nonvisceral (musculoskeletal and cutaneous) or visceral (neurological, renal, hematological, pulmonary, gastrointestinal, and cardiovascular). Systemic involvement occurs through two main mechanisms: periepithelial infiltration and extraepithelial immune complex-mediated damage [2].

Among the visceral manifestations, renal involvement most commonly presents as tubulointerstitial nephritis (TIN), caused by lymphocytic infiltration of the renal interstitium, which leads to tubular dysfunction [1]. A key clinical consequence of TIN is renal tubular acidosis (RTA), with distal RTA (dRTA) being the most frequently observed subtype, occurring in up to 20% of pSS patients [3]. dRTA is characterized by defective hydrogen ion secretion in the distal tubules, resulting in non-anion gap metabolic acidosis (NAGMA), persistently high urinary pH (>5.5), and hypokalemia. These disturbances can lead to muscle weakness, nephrocalcinosis, nephrolithiasis, and metabolic bone diseases such as osteomalacia and osteoporosis.

A classic manifestation of hypokalemic paralysis involves the acute onset of symmetrical, ascending proximal muscle weakness accompanied by reduced deep tendon reflexes, typically without any alteration in consciousness. Although osteomalacia is rarely the initial presentation of pSS, it can emerge as a long-term complication of chronic dRTA in affected individuals [4].

## Case presentation

A 42-year-old woman presented with a history of five similar episodes of flaccid quadriparesis over the last four years. Each episode had a sudden onset, was unrelated to carbohydrate-rich meals or physical exertion, and resolved completely after potassium supplementation. More recently, she had developed progressive difficulty in rising from a seated position and performing overhead activities over the preceding six months. Additionally, she reported groin pain and increasing difficulty walking over the last two months.

She denied any associated symptoms, such as fever, joint swelling or pain, dry eyes or mouth, skin rashes, Raynaud’s phenomenon, or muscle pain. A detailed clinical history was obtained to identify any potential contributing factors. She had no history suggestive of malabsorption, thyroid dysfunction, prolonged steroid use, alcohol or tobacco use, or self-induced vomiting. However, she did report an unintended weight loss of approximately 7 kg over the last six months.

On general examination, the patient appeared mildly anemic, with no signs of thyroid enlargement or lymphadenopathy. Neurological assessment revealed reduced muscle strength - Medical Research Council grade 3 out of 5 in proximal muscles and 4 out of 5 in distal muscles - in all four limbs, while all sensory modalities were preserved. Additionally, hip joint movement was restricted bilaterally.

Initial laboratory workup showed normocytic, normochromic anemia on CBC. Serum potassium was significantly low at 2.1 mmol/L. Arterial blood gas analysis revealed normal anion gap metabolic acidosis, with a bicarbonate level of 13.5 mmol/L and a positive urinary anion gap - findings consistent with a diagnosis of dRTA (Table 1).

**Table 1:** Laboratory investigations showing severe hypokalemic, hyperchloremic metabolic acidosis, along with elevated urinary pH and a positive urine anion gap, consistent with a diagnosis of dRTA ABG: arterial blood gas; dRTA: distal renal tubular acidosis; Hb: hemoglobin; MCH: mean corpuscular hemoglobin; MCV: mean corpuscular volume

Investigation	Result	Reference range
CBC		
Hb	9.3 gm/dl	11.20-15.70 gm/dl
MCV	97.8 fl	76-96 fl
MCH	29.3 pg	27-32 pg
RBC	3.17 million/dl	4.04-6.12 million/dl
Serum electrolytes		
Sodium	144 mmol/L	135-145 mmol/L
Potassium	2.1 mmol/L	3.5-5.0 mmol/L
Chloride	123 mmol/L	95-105 mmol/L
Bicarbonate	13.5 mmol/L	12-22 mmol/L
Anion gap	9.6 mmol/L	8-16 mmol/L
ABG		
pH	7.25	7.35-7.45
pCO₂	30.8 mmHg	38-42 mmHg
HCO₃⁻	13.5 mmol/L	22-28 mmol/L
Urine pH	7.25	7.35-7.45
Urine electrolytes		
Sodium	40 mmol/L	40-220 mmol/L
Potassium	20 mmol/L	25-125 mmol/L
Chloride	44 mmol/L	14-150 mmol/L

The patient underwent a comprehensive evaluation to identify potential causes of dRTA, including a detailed drug history and autoimmune screening. Serological analysis revealed positive antinuclear antibody (ANA), anti-SSA/Ro, and anti-SSB/La antibodies. Other autoimmune markers, including anti-dsDNA, anti-Smith antibody, and rheumatoid factor, were negative (Table 2). Objective evidence of glandular involvement was demonstrated by markedly reduced unstimulated salivary flow and Schirmer’s test results (3 mm in both eyes within five minutes) (Table 2). Additionally, salivary gland ultrasonography showed characteristic features consistent with Sjögren’s syndrome.

**Table 2:** Laboratory findings indicative of pSS ANA: antinuclear antibody; anti-SSA/Ro: anti-Sjögren’s syndrome-related antigen A; anti-SSB/La: anti-Sjögren’s syndrome-related antigen B; pSS: primary Sjögren’s syndrome; RA: rheumatoid arthritis

Investigation	Result	Reference range
ANA screening	Positive (>400)	<40 AU/mL
Anti-Ro/SS-A	Positive (>400)	0-40.0 AU/mL
Anti-La/SS-B	Positive (>195)	0-40.0 AU/mL
Unstimulated salivary flow	0.056 ml/min	<0.1 ml/min considered salivary gland hypofunction
Schirmer’s test	Rt eye 3 mm in five minutes; Lt eye 3 mm in five minutes	<5 mm in five minutes in at least one eye is considered dry eye
Anti-ds-DNA	Negative	10-15 IU/mL
RA	Negative	0-20 IU/mL

As the patient developed proximal myopathy, further investigations were conducted to rule out alternative etiologies. Laboratory tests revealed hypocalcemia (serum calcium 8.0 mg/dL), hypophosphatemia (serum phosphate 1.5 mg/dL), and elevated alkaline phosphatase (203 U/L). Additionally, 25-hydroxy vitamin D was markedly low at 10.3 ng/mL, confirming vitamin D deficiency. Bone mineral density assessments showed osteopenia in the lumbar spine and both femoral necks (Table 3). These findings were consistent with osteomalacia. Serum creatine phosphokinase and thyroid-stimulating hormone levels were within normal ranges (Table 3).

**Table 3:** Biochemical and imaging findings indicative of osteomalacia and osteopenia ALP: alkaline phosphatase; CPK: creatinine phosphokinase; DEXA: dual-energy X-ray absorptiometry; PTH: parathyroid hormone; TSH: thyroid-stimulating hormone

Investigation	Result	Reference range
CPK	Negative	30-135 U/L
S. TSH	1.84 mIU/L	0.7-1.3 mg/dl
S. calcium	8.0 mg/dl	8.5-10.2 mg/dl
S. inorganic phosphate	1.5 mg/dl	2.4-5.1 mg/dl
S. magnesium	2.1 mg/dl	1.82-2.30 mg/dl
S. ALP	203 U/L	40-150 U/L
PTH	113.5 pg/ml	10-65 pg/ml
S. inorganic phosphate	1.5 mg/dl	2.4-5.1 mg/dl
Vitamin D level	10.3 ng/ml	<20 ng/ml
DEXA scan	Lumbar first to fourth vertebrae: T score -2.0, Z score -1.6; right femoral neck: T score -1.8, left femoral neck: T score -1.2	

**Figure 1:**
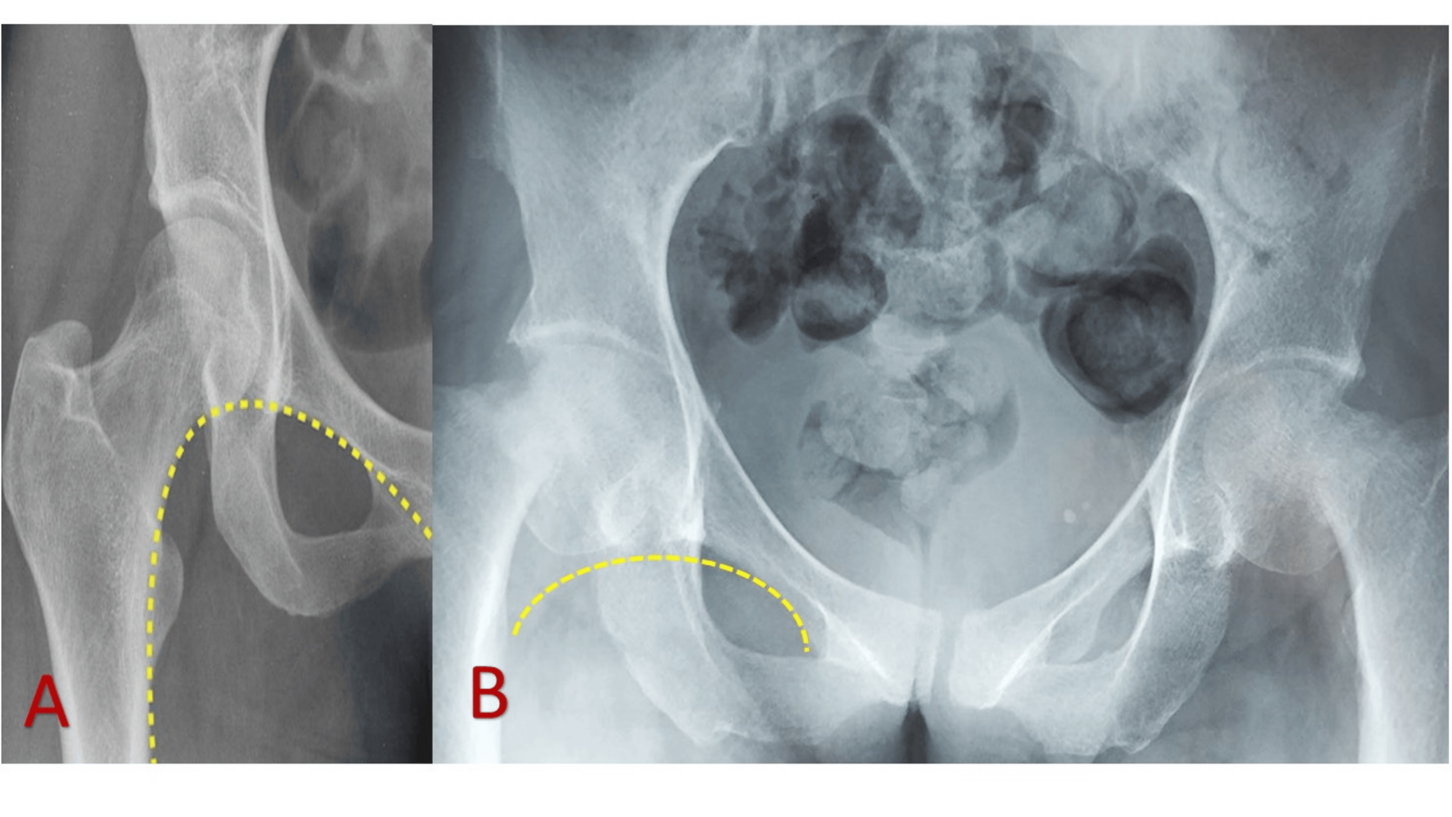
Plain X-ray of the pelvis and femur (AP view): (A) Preservation of the normal Shenton’s line with no significant findings. (B) Bilateral shortening of the femoral necks, reduced joint space, and disruption of Shenton’s line, indicative of old femoral neck fractures in a 42-year-old woman

A plain radiograph of the pelvis revealed bilateral old femoral neck fractures (Figure 1), indicative of bone complications associated with chronic dRTA.

The patient was started on potassium chloride syrup, 30 mL daily in three divided doses, providing a total of 40 mEq/day, along with sodium bicarbonate (500 mg), two tablets four times a day, delivering 12 mEq of bicarbonate with each 1000 mg dose. She was also prescribed weekly cholecalciferol for eight weeks and calcium supplementation. After one week of inpatient treatment, the patient showed significant improvement, with her potassium levels stabilizing between 3 and 3.5 mEq/L and experiencing minimal symptoms. She was discharged with instructions for close follow-up and scheduled for outpatient reevaluation. Additionally, she was referred to orthopedics for the management of her old femoral fractures.

## Discussion

Sjögren’s syndrome is an autoimmune disorder characterized by lymphocytic infiltration and inflammation of the exocrine glands, resulting in decreased glandular secretion. This typically manifests as dry eyes and dry mouth. The estimated prevalence of Sjögren’s syndrome is 10.3 per 10,000 people, with a notably higher occurrence in females, at a male-to-female ratio of 1:16. Extraglandular manifestations are observed in about one-third of cases, and renal involvement is reported in 4.9% of patients [5]. While glandular involvement usually precedes extraglandular symptoms, in some cases, the latter may present first. Diagnosing Sjögren’s syndrome can be particularly challenging when extraglandular symptoms appear initially [6].

Sjögren’s syndrome is classified into two forms: primary and secondary. pSS occurs as an independent condition, whereas secondary Sjögren’s syndrome develops in the context of other rheumatologic diseases, such as systemic lupus erythematosus, rheumatoid arthritis, or scleroderma [7]. The diagnosis of pSS is confirmed when a patient meets four out of six classification criteria, which include histopathological findings or the presence of autoantibodies. Alternatively, a diagnosis can be made if the patient fulfills three out of four objective criteria [8]. Our patient meets three out of four objective criteria.

Renal involvement, an extraglandular manifestation of Sjögren’s syndrome, affects less than 10% of cases. RTA is seen in 4.5-9% of patients, typically in middle-aged individuals, with only two-thirds of these patients being symptomatic. In pSS, kidney involvement is primarily due to TIN, with glomerular disease being less common [9]. Renal involvement in Sjögren’s syndrome can be classified into two types: peri-epithelial, caused by lymphocytic infiltration, and extra-epithelial, due to immune complex deposition. Peri-epithelial lesions affect the proximal tubules, intercalated cells, or the loop of Henle [10].

pSS can affect the entire nephron, leading to both proximal and dRTA. The most common clinical features of dRTA include NAGMA, alkaline urine, and hypokalemia. In this patient, the combination of NAGMA, hypokalemia, elevated urine pH, and a positive urine anion gap supports a diagnosis of dRTA with predominant renal potassium loss [11]. Distal RTA, often asymptomatic, is a frequent manifestation of Sjögren’s syndrome that remains undiagnosed in many cases. Hypokalemia is the most common electrolyte disturbance associated with dRTA, occurring in approximately 28-53% of patients. Notably, hypokalemia can precede the classic glandular symptoms of Sjögren’s syndrome, potentially facilitating an earlier diagnosis. In some cases, hypokalemic paralysis serves as the initial presentation, affecting around 7% of patients [9].

Distal RTA is characterized by defective hydrogen ion (H⁺) secretion in the distal nephron, impairing urinary acid excretion and reducing ammonium (NH₄⁺) excretion. To maintain electroneutrality, there is an increase in distal potassium excretion, leading to hypokalemia. Severe hypokalemia (<2.5 mmol/L) can cause acute flaccid paralysis, ranging from mild muscle weakness to profound paralysis [12].

Metabolic bone disease and osteomalacia due to dRTA in Sjögren’s syndrome have been previously documented. In distal RTA, chronic acidosis combined with hypophosphatemia contributes to bone demineralization [13]. Chronic metabolic acidosis suppresses both bone formation and resorption, leading to reduced bone mass. This abnormal bone remodeling is characterized by low-turnover bone disease with defective mineralization. Prolonged metabolic acidosis can impair osteoblast function and disrupt bone matrix mineralization, contributing to osteomalacia. Additionally, coexisting vitamin D deficiency may further exacerbate bone loss [14]. In our patient, clinical and biochemical findings were consistent with osteomalacia.

The treatment approach for Sjögren’s syndrome varies depending on the presenting symptoms and the presence of extraglandular manifestations. Hydroxychloroquine is the first-line treatment for systemic manifestations of Sjögren’s syndrome, including arthralgia, arthritis, and fatigue. For patients with severe systemic involvement, corticosteroids are the mainstay of therapy, often combined with immunosuppressive agents. While pSS generally follows a benign course, certain features such as vasculitis, glandular enlargement, low complement levels, and cryoglobulinemia are linked to a higher risk of severe complications, including non-Hodgkin lymphoma. The management of dRTA primarily focuses on supportive care, including potassium and bicarbonate supplementation, while monitoring for complications such as nephrolithiasis. Early diagnosis and long-term alkali treatment are crucial for preventing both acute hypokalemia and long-term complications like osteomalacia, kidney stones, and progression to chronic kidney disease [15].

## Conclusions

This case underscores the importance of considering pSS in middle-aged women presenting with recurrent hypokalemic quadriparesis, even in the absence of classic sicca symptoms. Hypokalemia may serve as an indicator of underlying renal involvement, particularly dRTA, which can lead to complications such as osteomalacia and osteoporosis. Early diagnosis and appropriate management are crucial to prevent long-term morbidity and improve patient outcomes.
